# Perceived outcomes of medical teaching institute reforms: insights from management, faculty, and administration in Pakistani tertiary health care

**DOI:** 10.1186/s12913-024-11416-y

**Published:** 2024-09-13

**Authors:** Wajiha Qamar, Mehran Qayum, Waqar-un Nisa, Asma Ali

**Affiliations:** 1 Department of Oral Biology, Bacha Khan College of Dentistry, Mardan, Pakistan; 2 Evidence for Health (E4H) Programme Khyber Pakhtunkhwa, Oxford Policy Management, Peshawar, Pakistan; 3 Department of Oral Pathology, Bacha Khan College of Dentistry, Mardan, Pakistan

**Keywords:** Health, Reforms, Tertiary Health Care, Pakistan, Service Delivery, Medical Education, Faculty

## Abstract

**Objective:**

The study aims to explore the perceived outcomes of Medical Teaching Institution (MTI) reforms on autonomy and overall performance within tertiary healthcare institutions in Khyber Pakhtunkhwa (KP) province, Pakistan.

**Methodology:**

A cross-sectional study was carried out from September 2023 to March 2024, involving interviews with frontline staff, administrative personnel, and senior management within MTI-affiliated institutions. The methodology employed, using both qualitative and quantitative data analysis techniques.

**Results:**

The study showed that institutional staff members’ knowledge and understanding of the MTI changes differed. Some observed very minor adjustments, while others saw advances in hospital operations and service delivery. Administrative complexity, political meddling, and resource allocation problems were noted as challenges. Positive results were also observed, though, and they included improved infrastructure, possibilities for staff training, and decision-making procedures.

**Conclusion:**

Despite significant improved, there are still challenges, such as inconsistent staff comprehension, mixed impacts on service delivery, resource allocation issues, and political meddling. Addressing these issues necessitates improved communication, continuous evaluation, and coordinated efforts to improve administrative systems and obtain consistent funding.

**Supplementary Information:**

The online version contains supplementary material available at 10.1186/s12913-024-11416-y.

## Introduction

In the constantly evolving field of public health, reforms are crucial phases that impact the course of healthcare systems and adapt to the evolving needs of the population [1–3]. To be successful in this effort, these reforms need to be carefully designed and tailored to the distinctive needs of the region [4]. Once in office, each government usually makes health its main objective and works to improve health through reforms; however, the implementation of these reforms frequently takes time, compelling governments and political leaders to actively seek innovative methods to elevate healthcare quality and accessibility in response to these pressing issues. [5] These strategies include efforts to lessen gaps in access to healthcare services, infrastructure development, regulatory reforms, and investments in healthcare technology.

Global reforms in medical teaching institutions and tertiary healthcare systems provide substantial perspectives into different ways of enhancing healthcare delivery and education. For example, the National Health Service (NHS) reforms in the United Kingdom aimed to decentralize management and promote competition by delegating public health responsibilities to local authorities [6]. In the United States, the Accreditation Council for Graduate Medical Education emphasized standardizing residency training, implementing competency-based education, and enhancing oversight while encouraging residents to balance their professional and personal lives [7]. The Diagnosis-Related Groups system, introduced in Germany, aims to restructure hospital financing in order to increase efficiency and quality [8]. Similarly, Canada’s CanMEDS system included competency-based courses, improved interprofessional education, standardized residency training, and better assessment procedures [9]. The National Medical Commission Act of 2019 in India created a new regulatory organization, imposed severe criteria for education and practice, implemented a national exit test for medical graduates, and tried to increase transparency and accountability [10]. Understanding these various global strategies provides an international perspective that enriches the analysis of Pakistan’s MTI reforms by providing valuable insights into how different approaches have shaped healthcare systems and medical education around the world, as well as informing the evaluation and refinement of Pakistan’s own reform efforts.

This pursuit of healthcare reform is not without its challenges. A dedication to surmounting financial obstacles, careful planning, and a focus on enhancing healthcare services and infrastructure are all necessary for the successful implementation of significant healthcare changes. Furthermore, it demands a comprehensive comprehension of the distinct socio-cultural and economic elements operating in every lower middle-income country. It involves acknowledging the numerous requirements and needs of communities and navigating the complex interactions between healthcare regulations and more general socioeconomic elements like employment, education, and poverty levels [11]. 

In the context of Pakistan, the country’s overall health expenditure for the 2019–20 fiscal year was Rs. 1,466 billion, indicating that the current health expenditures (CHE) to Gross Domestic Product (GDP) ratio was 3.0% [12]. The country’s annually per capita CHE were $40.7 US dollars, and the out-of-pocket (OOP) portion of the CHE was 54% [12]. This illustrates how the healthcare system is heavily dependent on household out-of-pocket expenses for funding. Pakistan employs a three-tiered basic, secondary, and tertiary healthcare system called the “Beveridge model”. The country aspires to achieve Universal Health Coverage (UHC) by 2030 and has made significant progress to enhance healthcare delivery through several initiatives [13]. But challenges still exist, especially in primary healthcare, where inadequate service delivery puts a significant strain on secondary and tertiary healthcare levels [13]. 

Prior to the MTI reforms, Pakistan’s healthcare system was fragmented and inadequately funded, resulting in inconsistencies in service quality and access. Medical education was outdated in date, lacked alignment with contemporary practices, and institutions had limited capacity to innovate or effectively meet local needs.

The government of Khyber Pakhtunkhwa (KP) province of Pakistan implemented several reforms over the years to commemorate a major turning point in the province’s healthcare landscape [14]. The Medical Teaching Institution (MTI) Act of 2015, a landmark set of legislation designed to completely transform public teaching hospitals in KP, served as the impetus for these changes [15]. The government that was at the helm at the time enacted the legislation, indicating its commitment to improve the standard of healthcare delivery. The MTI Act of 2015 was drafted and enacted as an essential component of this larger healthcare reform, initiating the huge restructuring effort.

The first stage of the reform was to provide public teaching hospitals more autonomy. This was a strategic measure meant to enhance the facilities’ overall effectiveness. The government aimed to promote positive improvements and empower public teaching hospitals to swiftly adapt to the evolving healthcare environment by granting them some degree of self-governance. The MTI Act’s autonomy was meant to promote innovation, provide administrators in hospitals more authority, and maximize resource utilization for better patient care.

In KP, the 10 tertiary healthcare hospitals designated under the MTI Act are undergoing reforms aimed at boosting their autonomy. The MTI Act empowers these institutions to manage their administration, finances, and academic matters independently, with the objective of increasing efficiency and responsiveness. By minimizing bureaucratic delays and fostering local innovation, the reforms aim to assist these hospitals in better addressing regional healthcare concerns and improving the quality of medical education and patient care.

Under the MTI Act, this transformative phase was managed using a multifaceted approach. One of the most significant components was the establishment of autonomous boards of governors, which took the place of the traditional civil bureaucracy in the administration of healthcare facilities [14]. These boards were given substantial decision-making power and administrative autonomy with the goal of improving overall effectiveness and streamlining processes.

The MTI Act recognised the critical importance of tertiary-level medical institutions as hubs for medical education, research, and specialised patient care, and it implemented structural adjustments to address these needs [16]. Through the implementation of amendments at this critical level, the Act aimed to address major concerns in healthcare. The intention of these reforms was to provide the institutions more autonomy and flexibility so that they could make important operational choices [16]. The act also aimed to establish new guidelines for professional development in these institutions by promoting promotions based on merit. Its objectives were to recruit and retain highly qualified medical personnel, raise the standard of education, and raise the general standard of healthcare services by implementing more transparent and performance-driven promotional practices [16]. 

But as with any major reform effort, there were significant issues highlighted by the KP MTI Act’s implementation. Even while it represented a significant departure from the conventional civil arrangement, its success in accomplishing the desired outcomes was still up for debate [17]. Because of this uncertainty, a thorough research into how the KP MTI Act of 2015 affected the autonomy and recruitment practices of Khyber Pakhtunkhwa’s medical teaching institutions was necessary [17]. 

This paper aims to bridge this research gap by conducting an in-depth review and offering insights from perspectives of management, academics, and administration inside those influenced institutions. By critically examining the outcomes and implications of the KP MTI Act, this article attempts to explain the nuances of the autonomy provisions specified in the MTI Act as it explores the success of the KP MTI reforms. The research attempts to add significant insights to the larger knowledge of healthcare transitions in low- and middle-income countries by critically analysing the objectives, management approaches, and results of this crucial change.

### Methodology

This cross-sectional study was conducted in an MTI institute to assess the effectiveness of the MTI reforms in improving autonomy and overall performance of the institutes that have implemented MTI. The MTI Act regulates the operations of ten MTI institutes within the province. Because the study was not funded by any academic institution, it was undertaken at only one MTI institute. Despite this limitation, the study’s findings remain valid because the researchers used an extensive methodology. The use of stratified random sampling and robust collecting data techniques ensured that the insights gathered were reliable and reflected the experiences of the selected institute. Furthermore, the results were contextualized within the larger framework of MTI reforms, which helped to validate and generalize the findings to other MTI institutes in the province.

The study spanned from September 2023 to March 2024, and was ethically approved from Bacha Khan College of Dentistry in Mardan on September 1st, 2023, after verifying that it complied with ethical guidelines. The study used a convergent mixed-methods approach, in which quantitative and qualitative data were collected concurrently but analyzed separately. The objective for this strategy was to take advantage of the strengths of both methodologies: the quantitative phase gave broad, measurable evidence of the MTI reforms’ impact, whereas the qualitative phase provided deeper context and explanations for the observed patterns. This design was used to ensure a thorough knowledge of the reforms from a variety of perspectives, as well as to improve the findings’ validity and depth. The study started with quantitative data collecting, utilizing a structured questionnaire to identify general patterns and trends. Following that, qualitative data was collected through open-ended interviews with the objective of delving deeper into participants’ perspectives. The data were integrated throughout the analysis phase, which assessed quantitative patterns as well as qualitative insights to provide a comprehensive understanding of the MTI reforms.

In this study, effectiveness is defined as the degree to which MTI reforms enhance the institute’s performance and outcomes. This involves improvements in operational efficiency, patient care quality, and adherence to reform objectives, as measured by through structured questionnaires. Autonomy is defined as the degree to which MTI institutes gained independence over their administrative and operational decisions. This involves the ability to make independent decisions about money allocation, staffing, and policy implementation with minimal outside interference.

The study population included three different participant groups: senior management, healthcare providers, and administrative personnel. The healthcare provider category consisted of doctors, nurses, and paramedical staff, while the administrative staff category encompassed individuals engaged in finance, administration, procurement, litigation, and logistics. The senior management group also included deans, associate deans, and principals, as well as representatives from the academic council. A list of all faculty members at the MTI institute who met the study criteria—having been employed at the MTI for at least three years and falling into one of three categories: senior management, health care providers, or administrative personnel—was prepared. Participants were categorized into three groups based on stratified random sampling. A simple random selection process was used in each stratum to pick 27 healthcare practitioners, 6 senior management team members, and 6 administrative persons for interviews. Prior to participation, all prospective participants were given an in-depth explanation of the ethical criteria to ensure informed consent and data confidentiality.

To address the research questions from different viewpoints, we developed both quantitative and qualitative tools. The quantitative instrument was a customized self-structured questionnaire developed to assess the effectiveness of various MTI reforms. This questionnaire was developed after doing an in-depth review of the literature and was pilot tested on a small sample size to identify possible challenges and ensure clarity. A group of experts assessed the revised tool to ensure that it addressed all key areas, and their feedback, combined with pilot test results, drove the final adjustments. The data collection process included both structured and open-ended questions. Open-ended interviews were used in the qualitative stage to delve deeply into the perspectives of participants. The method used for these interviews involved recording, transcribing, and categorizing responses to find patterns and themes. To extract useful insights from the qualitative data, thematic analysis was used. The quantitative phase involved tallying, cross-checking, and analyzing data from interviews using appropriate statistical techniques in Excel. For the qualitative part of the study, open-ended questionnaires were developed to delve deeply into participants’ perspectives. The interviews were intended to supplement the quantitative questionnaire by examining areas that needed more explanation. These tools were also improved through pilot testing and expert review to ensure that they acquired rich, detailed responses from participants.

The analytical framework analyzed the data using both sequential and integrative techniques. Initially, quantitative data were analyzed in Excel using statistical techniques, leading to an overview of the MTI reforms’ impact and the identification of major trends and patterns. The qualitative data from open-ended interviews were then collected, transcribed, and categorized to discover themes and gain specific insights into participants’ opinions. Integrating these analyses provided a holistic picture of the study’s findings, combining broad quantitative trends with detailed qualitative context. The findings of the study are presented in a structured manner reflecting the explanatory design of the study.

## Results

To assess the effectiveness of MTI reforms, a three-pronged approach was used. Data were gathered from health care providers, administrative staff, and senior management to provide an in-depth analysis. The results of both quantitative and qualitative analyses are integrated into all areas of study to provide a comprehensive overview of the study’s findings.

### Awareness and comprehension of MTI reforms

Most healthcare providers (*n* = 18, 62.07%) were aware of the MTI reforms, yet they exhibited insufficient knowledge about the specifics and implications of the reforms. Instead, they were mostly ambiguous with respect to the reforms’ particular provisions, focusing on issues such as appointments, procurement, and revenue generation. A professor in the Clinical Sciences department noted, “The MTI institution includes the roles of a dean, hospital director, and medical director,” while a nurse added, “It refers to easy appointment, procurement, and revenue generation for the institute.”

Administrative staff demonstrated varying levels of awareness, with only half claiming a thorough understanding of the MTI reforms. They saw the MTI as a delegation of power, albeit some were unaware of the exact legislation.

In contrast, senior management demonstrated a thorough comprehension of the reforms and their intended outcomes. A faculty member pointed out, “The Board of Governors (BOGs) acts as the institute’s decision-making body, giving it the ability to make decisions on its own and manage finances and income.”

### Impact on institutional management and service delivery

Healthcare providers perspectives on the impact of the MTI reforms on institutional management and service delivery differed. Some healthcare providers observed efficiency gains (*n* = 6, 20.69%), while others saw no meaningful difference (*n* = 15, 51.72%). A professor mentioned “Yes, improvement has been seen since the implementation of MTI reform,” while a nurse commented, “multi-disciplinary services are now available in hospitals.”

Administrative staff reported no substantial reforms in administrative procedures (*n* = 5, 83.33%). They emphasized improvements in decision-making and fund release, but also criticized a lack of clear direction and budget allocation concerns. Senior management cited significant improvements in institutional management, particularly in financial management and decision-making procedures. An academic council member commented, “The institution has experienced an increase in the utilization of cutting-edge technology and infrastructure investments.”

### Quality of patient care

In terms of patient care quality, 48.15% (*n* = 13) of healthcare providers saw an improvement, but 51.85% (*n* = 14) did not. Many participants mentioned the use of innovative technology and the availability of senior clinicians around the clock. A nurse on duty stated: “The introduction of institutional-based practices has significantly increased patient care.” However, some individuals identified malpractices that inhibit high-quality services. An associate professor said: “Patients are often directed to evening sessions for institutional-based practices instead of the Outpatient Department (OPD), where they are charged fees.”

Administrative staff reported similar improvements, but they also mentioned challenges including increasing workload and resource allocation challenges. Senior management noted improvements in patient care due to enhanced infrastructure and technology but emphasized the importance of maintaining a focus on quality.

### Resource allocation and utilization

Concerning resource distribution, 48.15% (*n* = 13) of healthcare providers were satisfied with the practices, while 51.85% (*n* = 14) were not. Improvements noted were the provision of new equipment and the excellent control of material waste. However, issues such as a scarcity of available resources and uncertainty about resource availability were identified. According to a lecturer, “Most of our dental units are not functional.”

Administrative staff also had mixed experiences with resource distribution, with 66.67% (*n* = 4) indicating improvements. They emphasized how easy it is to release funds for MTI projects. A staff nurse said, “It has become easier and more feasible to release finances for a project under MTI.” Senior management emphasized improved resource allocation while acknowledging reliance on government money and political meddling.

### Collaboration among healthcare professionals

One third of the of healthcare practitioners 37.93% (*n* = 11) recognised improved collaboration among healthcare professionals, whereas 41.38% (*n* = 12) reported no notable improvement. Participants noted organising a variety of workshops and seminars, as well as implementing collaborative activities. However, administrative staff reported a lack of formal collaboration and coordination across several groups. Senior management emphasised the importance of actual autonomy and less political influence to improve teamwork.

### Training and professional development

Training and professional development opportunities improved for 62.07% (*n* = 17) of healthcare providers, while 34.48% (*n* = 10) reported no apparent improvements. A technician stated, “Trainings are taking place, but only for a select few staff members.” Administrative staff cited improvements in staff development and training opportunities but observed a lack of programs for administrative staff. Senior management recognized improvements in training programs but emphasized the importance of more inclusive access.

### Preferences and recommendations

Some healthcare practitioners favored the prior civil system, claiming increased resource availability and employment stability. A lecturer stated, “The previous civil system was better, as my job was secure; now it is not.” Administrative staff recommended revising the old method to address job security issues and improving accountability. Senior management emphasized the importance of clear norms and regulations, minimal political intrusion, and balancing autonomy with accountability.

### Administration and financial management

In terms of administrative and financial management, 66.67% (*n* = 4) of participants reported better financial processes, while 33.33% (*n* = 2) saw no major difference. A cashier stated, “The ease of releasing finances has improved.” Senior management acknowledged significant improvements in financial management, but also emphasized obstacles such as political interference and delayed payments of wages. One intriguing aspect that surfaced was that “The true autonomy doesn’t prevail, there is a great deal of political interference, the board dissolves when the health department’s top management changes, and it takes time for the new to become familiar with every detail. The institute still depends on government funding and does not generate sufficient of it; the political turmoil and switching places between the federal and provincial governments has hindered the flow of finances and disbursements, delayed salary payments and jeopardizing several initiatives.

## Discussion

The study presented multifaceted insights into the effectiveness of the MTI reforms in enhancing autonomy and overall performance. We found high awareness but varied comprehension of the MTI reforms among institutional staff. While the reforms were generally acknowledged by most participants, specific provisions were understood to a lesser extent, with senior management demonstrating greater awareness. This finding is consistent with other research showing that awareness does not always equate to thorough comprehension [18]. Inadequate clarity regarding the reform measures may impede their efficient implementation. Staff members may find challenging to align their actions and behaviours with the intended aims of the reform if they do not fully understand them, which might compromise the reforms’ efficacy. Overall staff buy-in and comprehensive comprehension are essential to the successful implementation of reforms [11]. 

Participants’ opinions on how the MTI reforms affected hospital operations and service delivery were not unanimous. Some reported increased effectiveness and higher-quality patient care, while others saw just slight changes. Healthcare reforms can result in differing outcomes due to several factors [15]. These factors might be differences socioeconomic differences across areas, and regional variances in the healthcare infrastructure. Research has highlighted the importance of factors like political will, institutional capacity, community involvement, and patient-centred approaches, emphasizing how small-scale initiatives can lead to system-wide reforms [19]. Addressing these approaches require taking into account local settings and prioritise areas in need of development is necessary to address these variances. Additionally, the necessity of ongoing monitoring and assessment of healthcare reforms in order to determine their efficacy and focus areas that require improvement [2, 15, 19]. This is especially pertinent to the MTI reforms in KP, since continued assessment may assist healthcare managers and legislators in improving plans and tackling new issues.

The research revealed both advantages and disadvantages in the allocation and utilisation of resources after MTI adjustments. While improvements in infrastructure and equipment supply were emphasized by the participants, challenges like compromised clinical staff and delayed resource availability were also mentioned. The ad hoc arrangements and inadequate resources hinder the flourishing of reforms in healthcare, highlighting the necessity for fundamental reform, as piecemeal solutions are inadequate to address the underlying challenges [19, 20]. Prior study demonstrates that in order to effectively reform healthcare, significant structural and systemic changes are required, while small-scale fixes are insufficient to solve the current health issues [2]. It is imperative to address resource constraints and optimize utilization to ensure equitable access to high-quality healthcare services.

Opinions on collaboration among healthcare professionals were mixed; some felt it had improved, while others saw no appreciable shift. Integrated and patient-centred care, as evidenced by literature hinges on effective teamwork, which has demonstrated positive effect on patient safety and outcomes [21]. This highlight the necessity of promoting a collaborative culture through programmes like interprofessional education and team-based care approaches.

Many participants acknowledged satisfaction for the improvements in professional development and training opportunities, but there were also complaints about the emphasis on senior faculty members and the dearth of training for other staff groups. Maintaining workforce competency and guaranteeing the provision of high-quality healthcare depends on training and professional growth [22]. Targeted interventions that provide an emphasis on ongoing learning for all staff categories—including technicians, nurses, and administrative staff—are necessary to address discrepancies in training opportunities.

The participants’ opinion on how the MTI changes affected administrative procedures and autonomy were not unanimous. While some noted increases in autonomy and decision-making processes, other issues were noted, including an overwhelming workload and a reliance on board approvals. Studies assessing hospital governance innovations have noted comparable difficulties [23]. Optimising autonomy and promoting organisational performance require strengthening administrative capability, reducing procedures, and improving accountability systems.

The introduction of new technology, improvements in infrastructure, and improved staff training opportunities as reported by the participants are in line with research looking at healthcare reforms in different contexts [2, 4]. These encouraging results highlight how MTI could enhance healthcare institutions and enhance workforce capacity, contributing to improved patient care and satisfaction.

However, challenges related to resource allocation, political interference, and administrative complexities are consistent themes across multiple studies [4, 15, 16]. Our findings echo concerns raised in previous research regarding the dependency on government financing, delays in budget approvals, and the disruptive impact of political transitions on institutional stability [5, 15]. These challenges underscore the complex nature of healthcare reforms and the need for multifaceted solutions. Addressing challenges requires collaborative efforts involving policymakers, healthcare providers, and other stakeholders to enhance accountability mechanisms, mitigating political interference, and investing in workforce development and infrastructure [15]. 

Even though this case study focusses on only one institute, the findings provide useful insights that can be applied to other MTIs in KP province or potentially across Pakistan. MTI reforms have demonstrated both promise and challenges in the context of tertiary healthcare in KP, Pakistan. While there have been substantial improvements, recurring challenges underline the importance of continual analysis and adaptive measures. The similarities in institutional architecture, governance procedures, and the sociopolitical climate in which these MTIs operate indicate that comparable problems and victories are likely to occur elsewhere.

Additionally, this study provides a strong foundation for other MTIs to enhance their reform processes by emphasizing critical aspects such as the significance of full staff buy-in, effective resource allocation, and continual reform assessment. Future research should look into longitudinal impacts and comparison studies with other regions to have a better understanding of the reforms’ effectiveness. Policymakers should prioritize building a conducive climate for reform implementation, ensuring enough resources, and cultivating a culture of continual improvement. It also contributes to the field by giving empirical evidence on the complexity of healthcare reform as well as insights into potential avenues for improving the success of such projects worldwide. As a result, the findings of this study can help to shape broader strategies for implementing and evaluating healthcare reforms across the area, promoting systemic improvements and cultivating a culture of excellence in medical education and healthcare delivery across the country.

## Conclusions

Although the MTI reforms have resulted in certain positive change, it is imperative to address the recognised barriers and inequalities to ensure the long-term success and sustainability of healthcare reforms in KP. The authors conclude that:

1. Staff are aware of the MTI reforms, but their comprehension varies. Senior management have a thorough understanding, whereas healthcare providers and staff members exhibit less clarity. This highlights the necessity of increased communication and training in ensuring successful change implementation.
2. The MTI reforms have had a mixed impact on institutional management and service delivery, with varying assessments of increased efficiency and patient care quality. While some healthcare professionals reported favorable changes, a large proportion reported minimal impact, emphasizing the importance of continual monitoring and adaptive tactics.
3. While the reforms enhanced infrastructure and equipment provision, challenges such as limited resources and delayed availability remain. Addressing these concerns is critical for ensuring equal access to high-quality healthcare services.
4. Training opportunities have expanded, notably for senior faculty, although discrepancies persist among other staff categories. Providing comprehensive access to ongoing learning is critical for retaining workforce capability.
5. The reforms have had an uneven impact on collaboration, with some improvements acknowledged but no major shift reported by others. Establishing a collaborative culture through interprofessional education and team-based treatment practices is critical for improving patient outcomes.
6. Although autonomy and decision-making improved, issues such as increased effort and dependency on board approvals were also identified. Improving administrative capacity and accountability systems is critical for maximizing organizational effectiveness.
7. Persistent problems with political involvement and dependency on funding from the government have been discovered, affecting institutional stability and financial management. Collaborative efforts to reduce political interference and secure consistent funding are critical for maintaining reform benefits.

## Electronic supplementary material

Below is the link to the electronic supplementary material.

Supplementary Material 1

## Data Availability

The datasets generated and/or analyzed during the current study are not publicly available because we assured all participants that their data would be handled with the utmost confidentiality and would not be shared with any other parties. However, these datasets are available from the corresponding author upon reasonable request.

## Declarations

### Disclaimer

The views expressed in this paper represent the authors’ opinions based on their study and do not reflect the views of their associated institute/ organization.
