# Supplementary material for: Perceived outcomes of medical teaching institute reforms: insights from management, faculty, and administration in Pakistani tertiary health care
Source: BMC Health Serv Res. 2024 Sep 13;24:1061. doi: 10.1186/s12913-024-11416-y (PMC11396421; doi:10.1186/s12913-024-11416-y)
Supplement: Supplementary file 1 — Supplementary Material 1 [file 12913_2024_11416_MOESM1_ESM.docx]

| Thank you for participating in this study. The authors are dedicated to preserving the confidentiality of your identity. Your individual identity will remain undisclosed, and the information you provide will be aggregated for presentation in group data. The raw, individual information will not be shared with anyone.  The data collected will be used for publication and shared with decision-makers to improve MTI reforms and generate evidence for policymaking. Your responses will be presented as group data in research, and we assure you that your confidentiality will be maintained.  By agreeing to this, you give your consent to participate in this study. Participation is voluntary, and you may withdraw from the interview at any point if you feel uncomfortable or do not wish to continue.  Your participation is invaluable and will significantly contribute to advancing knowledge in this field. |
| --- |

## **Administration Department**

1. Demographic Information:
   1. Name:
   2. Department:
   3. Position:
   4. Years of service at the MTI institute:
   5. Gender:
   6. Age:
2. Familiarity with MTI Reforms: a.
   1. Are you familiar with the provisions of the MTI act in KP?
      1. Yes
      2. No
   2. Please provide a brief summary of the key provisions of the MTI act that you are aware of……………………………………………………………………………………………………………………………………..

…………………………………………………………………………………………………………………………………………

1. Impact on Administrative Processes:
   1. How has the MTI act affected the administrative processes within the institute?
   2. Improved efficiency
   3. Increased bureaucracy
   4. No significant change
2. Have you observed any changes in the decision-making authority and processes since the implementation of the MTI act? Please explain……………………………………………………………………………..

……………………………………………………………………………………………………………………………………………………..

1. Autonomy and Decision-Making:
   1. Has the MTI act provided increased autonomy in administrative decision-making?
      1. Yes
      2. No
   2. If yes, please provide specific examples of how the MTI act has enhanced autonomy in administrative processes………………………………………………………………………………………………….

…………………………………………………………………………………………………………………………………………

- 1. If no, please describe any challenges or limitations you have faced in exercising autonomy under the MTI act…………………………………………………………………………………………………………….

…………………………………………………………………………………………………………………………………………

1. Institutional Context:
   1. How has the institutional context changed since the implementation of the MTI act?
      1. Improved
      2. Declined
      3. No significant change
   2. In what ways has the MTI act influenced the effectiveness, efficiency, and responsiveness of the institute's administrative functions? Please provide specific examples…………………..

…………………………………………………………………………………………………………………………………………

1. Staff Development and Training:
   1. Have there been any improvements in staff development and training opportunities under the MTI act?
      1. Yes
      2. No
   2. If yes, please describe any specific changes or initiatives that have been implemented……
   3. If no, please explain any challenges or limitations you have observed in staff development and training since the implementation of the MTI act……………………………………………………….
2. Finance-related Questions:
   1. Has the MTI act impacted the financial management and budgeting processes within the institute?
      1. Yes
      2. No
   2. If yes, please describe any changes or improvements you have observed in the financial management and budgeting processes since the MTI act was implemented…………………….

…………………………………………………………………………………………………………………………………………

- 1. If no, please explain any challenges or limitations you have faced in financial management and budgeting under the MTI act……………………………………………………………………………………..

…………………………………………………………………………………………………………………………………………

1. Has the MTI act resulted in better utilization of financial resources for the institute's development and growth?
   - 1. Yes
     2. No
     3. If yes, please provide specific examples of how the MTI act has facilitated better utilization of financial resources…………………………………………………………………………..

…………………………………………………………………………………………………………………………….

- - 1. If no, please describe any challenges or limitations you have encountered in the utilization of financial resources under the MTI act……………………………………………….

…………………………………………………………………………………………………………………………….

1. Challenges:
   1. What challenges or limitations, if any, have you faced as an administrative staff member since the implementation of the MTI act?............................................................................

…………………………………………………………………………………………………………………………………………

1. Comparison with Previous System:
   1. If you were part of the administration staff before the MTI act was enacted, how does the current situation compare to the previous system in terms of administrative processes, autonomy, and staff development opportunities? (Open-ended)
2. Suggestions for Improvement:
   1. Based on your experiences and observations, what specific changes or improvements do you recommend to enhance the effectiveness of the MTI act in addressing the needs and concerns of the administrative staff?...................................................................................

…………………………………………………………………………………………………………………………………………

1. Is there any additional information or feedback you would like to provide regarding the MTI act and its impact on the administration staff?.......................................................................................

……………………………………………………………………………………………………………………………………………………..

## **Hospital staff**

1. Demographic Information:
   1. Name:
   2. Department:
   3. Position:
   4. Years of service at the affiliated teaching hospital:
   5. Gender:
   6. Age:
2. Familiarity with MTI Reforms:
   1. Are you familiar with the provisions of the MTI act in KP?
      1. Yes
      2. No
   2. Please provide a brief summary of the key provisions of the MTI act that you are aware of…………………………………………………………………………………………………………………………………….

………………………………………………………………………………………………………………………………………..

1. Impact on Hospital Operations:
   1. How has the implementation of the MTI act affected the overall operations of the affiliated teaching hospital?
      1. Improved efficiency
      2. Enhanced patient care
      3. Better resource allocation
      4. No significant change
      5. Other (Please specify……………………………………………………………………………………………
2. Can you provide specific examples or observations of how the MTI act has influenced hospital operations? ………………………………………………………………………………………………………………………………….

……………………………………………………………………………………………………………………………………………………..

1. Quality of Patient Care: a. In your opinion, has the MTI act contributed to an improvement in the quality of patient care at the affiliated teaching hospital?
   - 1. Yes
     2. No
     3. Not sure
        - If yes, please describe any specific changes or initiatives that have been implemented to enhance patient care……………………………………………….…..

……………………………………………………………………………………………………………….

- - - - If no, please explain any challenges or limitations you have observed in providing quality patient care since the implementation of the MTI act. ……..............................................................................................................

……………………………………………………………………………………………………………….

1. Resource Management:
   1. Have you noticed any changes in the allocation and utilization of resources within the hospital since the implementation of the MTI act?
      1. Yes
      2. No
         - If yes, please describe any specific changes or improvements you have observed in resource management………………………………………..……………..
         - If no, please explain any challenges or limitations you have faced in managing resources under the MTI act…………………………………………………..
2. Collaborative Approach:
   1. Has the MTI act encouraged a more collaborative approach among healthcare professionals at the affiliated teaching hospital?
      1. Yes
      2. No
      3. Not sure
         - If yes, please describe any specific examples of enhanced collaboration among different healthcare professionals……………………………………………….

……………………………………………………………………………………………………………….

- - - - If no, please explain any challenges or limitations you have encountered in fostering a collaborative environment since the implementation of the MTI act……………………………………………………………………………………………………

……………………………………………………………………………………………………………….

1. Training and Professional Development: a. Have there been any improvements in training and professional development opportunities for hospital staff since the MTI act was implemented?
   - 1. Yes
     2. No
        - If yes, please describe any specific changes or initiatives that have been implemented………………………………………………………………………………………….

……………………………………………………………………………………………………………….

- - - - If no, please explain any challenges or limitations you have observed in accessing training and professional development since the implementation of the MTI act……………………………………………………………….

……………………………………………………………………………………………………………….

1. Challenges:
   1. What challenges or limitations, if any, have you faced as a hospital staff member since the implementation of the MTI act?........................................................................................
2. Comparison with Previous System:
   1. If you were working at the hospital before the MTI act was enacted, how does the current situation compare to the previous system in terms of hospital operations, patient care, resource management, and collaboration among healthcare professionals? …………………………………………………………………………………………………………………………………………

…………………………………………………………………………………………………………………………………………

1. Based on your experiences and observations, what specific changes or improvements do you recommend to enhance the effectiveness of the MTI act in addressing the needs and concerns of the hospital staff and improving patient care?...................................................................................

……………………………………………………………………………………………………………………………………………………..

……………………………………………………………………………………………………………………………………………………..

## **Senior Management Team**

1. Demographic Information:
   1. Name:
   2. Position:
   3. Years of service in the MTI institute:
   4. Gender:
   5. Age:
2. Implementation Process:
   1. Can you provide an overview of the implementation process of the MTI act in the institute?
   2. What were the key steps taken to transition to the new system?
3. Impact on Autonomy and Decision-Making:
   1. How has the MTI act impacted your autonomy and decision-making authority in your role?
      1. Increased autonomy
      2. No significant change in autonomy
      3. Decreased autonomy
         - If yes, please provide specific examples of how the MTI act has enhanced or hindered your autonomy and decision-making processes………………….
4. Challenges and Concerns:
   1. What are the main challenges or concerns you have encountered as senior management and technical staff since the implementation of the MTI act?
   2. How are these challenges related to the provisions or implementation of the MTI act? …………………………………………………………………………………………………………………………………………

…………………………………………………………………………………………………………………………………………

1. Institutional Performance:
   1. In your opinion, how has the MTI act influenced the overall performance and functioning of the institute?
   2. Can you provide specific examples or observations of improvements or challenges in the institute's performance attributed to the MTI act?.............................................................

…………………………………………………………………………………………………………………………………………

1. Collaboration and Coordination:
   1. How has the MTI act influenced collaboration and coordination among different stakeholders, such as faculty, hospital staff, administration, and technical staff, within the institute?
   2. Please provide specific examples of collaborative initiatives, challenges faced, or areas of improvement under the MTI act……………………………………………………………………………………..
2. Financial Management:
   1. How has the MTI act impacted financial management and resource allocation within the institute?
   2. Can you provide specific examples of changes or challenges observed in financial management under the MTI act?.........................................................................................

…………………………………………………………………………………………………………………………………………

1. Utilization of Technical Expertise and Innovation:
   1. Has the MTI act encouraged the utilization of technical expertise and innovation within the institute? If yes, please provide specific examples……………………………………………………..

…………………………………………………………………………………………………………………………………………

1. Staff Development and Training:
   1. Have there been any improvements in staff development and training opportunities since the MTI act was implemented? If yes, please describe any specific changes or initiatives.
2. Based on your experiences and observations, what specific changes or improvements do you recommend to further enhance the effectiveness of the MTI act and address any challenges or concerns identified?...........................................................................................................................

……………………………………………………………………………………………………………………………………………………..

1. If you were part of the senior management or technical staff before the MTI act was enacted, how does the current situation compare to the previous system in terms of decision-making, autonomy, collaboration, institutional performance, financial management, utilization of technical expertise, and staff development?....................................................................................

…………………………………………………………………………………………………………………………………………………….

1. Is there any additional information or feedback you would like to provide regarding the MTI act and its impact on the senior management, technical staff, and the overall functioning of the institute?............................................................................................................................................

……………………………………………………………………………………………………………………………………………………..
